# Distinct phenotypic traits of *Staphylococcus aureus* are associated with persistent, contagious bovine intramammary infections

**DOI:** 10.1038/s41598-018-34371-1

**Published:** 2018-10-29

**Authors:** Tom Grunert, Beatrix Stessl, Franz Wolf, Daniel O. Sordelli, Fernanda R. Buzzola, Monika Ehling-Schulz

**Affiliations:** 1 Functional Microbiology, Institute of Microbiology, Department of Pathobiology, University of Veterinary Medicine, Vienna, Austria; 2 Institute of Milk Hygiene, Milk Technology and Food Science, Department for Farm Animals and Veterinary Public Health, University of Veterinary Medicine, Vienna, Austria; 3 Veterinarian Dr. Franz Wolf, Bad Schallerbach, Austria; 4 Instituto de Microbiología y Parasitología Médica (IMPaM), Universidad de Buenos Aires and CONICET, Buenos Aires, Argentina

## Abstract

*Staphylococcus aureus* causing persistent, recurrent bovine intramammary infections are still a major challenge to dairy farming. Generally, one or a few clonal lineages are predominant in dairy herds, indicating animal-to-animal transfers and the existence of distinct pathotypic traits. The aim of this study was to determine if long term persistence and spreading of *S. aureus* are associated with specific phenotypic traits, including cellular invasion, cytotoxicity and biofilm formation. Mastitis isolates were collected over a 3-years period from a single dairy herd, resulting in two persistent subtypes, the high within-herd prevalent subtype ST9 (CC9)-methicillin-susceptible *S. aureus* (MSSA), designated HP/ST9, and the low within-herd prevalent subtype ST504 (CC705)-MSSA, designated LP/ST504. Characterization of the two different coexisting persistent subtypes showed that the following phenotypic traits are particularly associated with high within-herd prevalence: lack of capsular polysaccharide expression, high cellular invasiveness, low cytotoxicity and high biofilm/ poly-N-acetylglucosamine (PNAG) production, which may concomitantly contribute to the spreading of HP/ST9 within the herd. By contrast to HP/ST9, LP/ST504 is characterized by the formation of colony dendrites, which may help the bacteria to access deeper tissues as niches for persistence in single animals. Thus, within a single herd, two different types of persistence can be found in parallel, allowing longtime persistence of *S. aureus* in dairy cattle. Furthermore, this study indicates that ST9 (CC9)-MSSA strains, which are currently thought to have their primary reservoir in swine and humans, can also successfully spread to new hosts and persist in dairy herds for years.

## Introduction

*Staphylococcus aureus* frequently causes intramammary infections (IMI) in dairy cattle. Most cases are chronic, persistent IMI, which are difficult to treat and prone to resurgence, and thus often accompanied by long-lasting cost intensive antibiotic treatment and premature culling^1,2^. Besides negative consequences for animal welfare and milk quality, *S. aureus* presents also a significant public health threat due to its zoonotic potential and risk of foodborne intoxications in humans^3,4^.

One of the most important aggravating factors of bovine *S. aureus* IMI is the capability of the bacteria to evade clearing by antibiotics and by the host immune system resulting in long-lasting persistent infections. Several phenotypic strain characteristics have been suggested to be linked to *S. aureus* long-term persistence in the mammary gland, including the capacity to form biofilms and to invade and/or survive intracellularly. Bacterial biofilms are recalcitrant to antibiotic treatment and host immune defense mechanisms^5,6^. The biofilm matrix comprises a complex mixture of several extracellular polymeric substances, such as polysaccharides, proteins and DNA. The polymeric N-acetylglucosamine (PNAG) is the main exapolysaccharide of the *S. aureus* biofilm matrix, which mediates the bacteria-to-bacteria interactions after primary attachment^7^. *S. aureus* is commonly recognized as an extracellular pathogen, but it is becoming increasingly evident that it can survive and even replicate inside non-professional as well as professional phagocytes^8,9^. The isolation of viable intracellular *S. aureus* from alveolar cells and macrophages derived from milk of chronically infected cows underscored the *in vivo* relevance of this niche^10^. Since several studies have shown a high prevalence of non-encapsulated strains in persistent bovine IMI (up to 86%)^4,11^ as well as human chronic infections^12^, loss of capsular polysaccharide (CP) expression may be a key feature associated with chronicity of *S. aureus*. It was reported that capsule loss promotes adherence and internalization of *S. aureus in vitro*^13,14^. A recent study of Bardiau *et al*. revealed a correlation of biofilm formation, low CP expression and cellular invasion, fostering the hypothesis that certain *S. aureus* strains might be associated with specific intracellular and extracellular niches within the host^15^. However, hitherto knowledge on particular strain characteristics allowing *S. aureus* to persist and spread within herds remains largely unknown.

*S. aureus* IMI often results in one or a few dominant clones within a herd indicating transmissibility between animals and the preference of particular pathotypic traits^16,17^. Recent studies indicate that high within-herd prevalence of *S. aureus* is linked to specific genotypes^18^. To gain further insights into the association of *S. aureus* within-herd prevalence with phenotypic and genotypic strain properties, we monitored *S. aureus* in a dairy herd for the period of three years by means of Fourier transform infrared (FTIR) spectroscopy. FTIR spectroscopy was employed since it is a biophotonic based method of high discriminatory power^19^, which was shown to a be a very suitable method for *S. aureus* subtyping^20^, allowing to track specific *S. aureus* biotypes within the dairy chain^4^. For our current study, two different *S. aureus* subtypes isolated from the same dairy herd, which were able to persist for years but differed in their within-herd prevalence, were selected. Genotypic and phenotypic properties associated with persistence, including cellular invasion, biofilm formation and cytotoxicity of these two *S. aureus* subtypes were investigated to unravel the mechanisms contributing to persistence and spread of *S. aureus* in herds of dairy cattle.

## Results

*S. aureus* persistence and spreading in a single dairy herd was monitored over a period of three years. Twelve cows out of in average 25 lactating cows were at least once positively tested for *S. aureus*. A total of 58 *S. aureus* isolates were submitted to FTIR spectroscopic and genotypic subtyping.

### The high IMI prevalent and persistent *S. aureus* isolates belong to ST9 (CC9)

**Figure 1:**
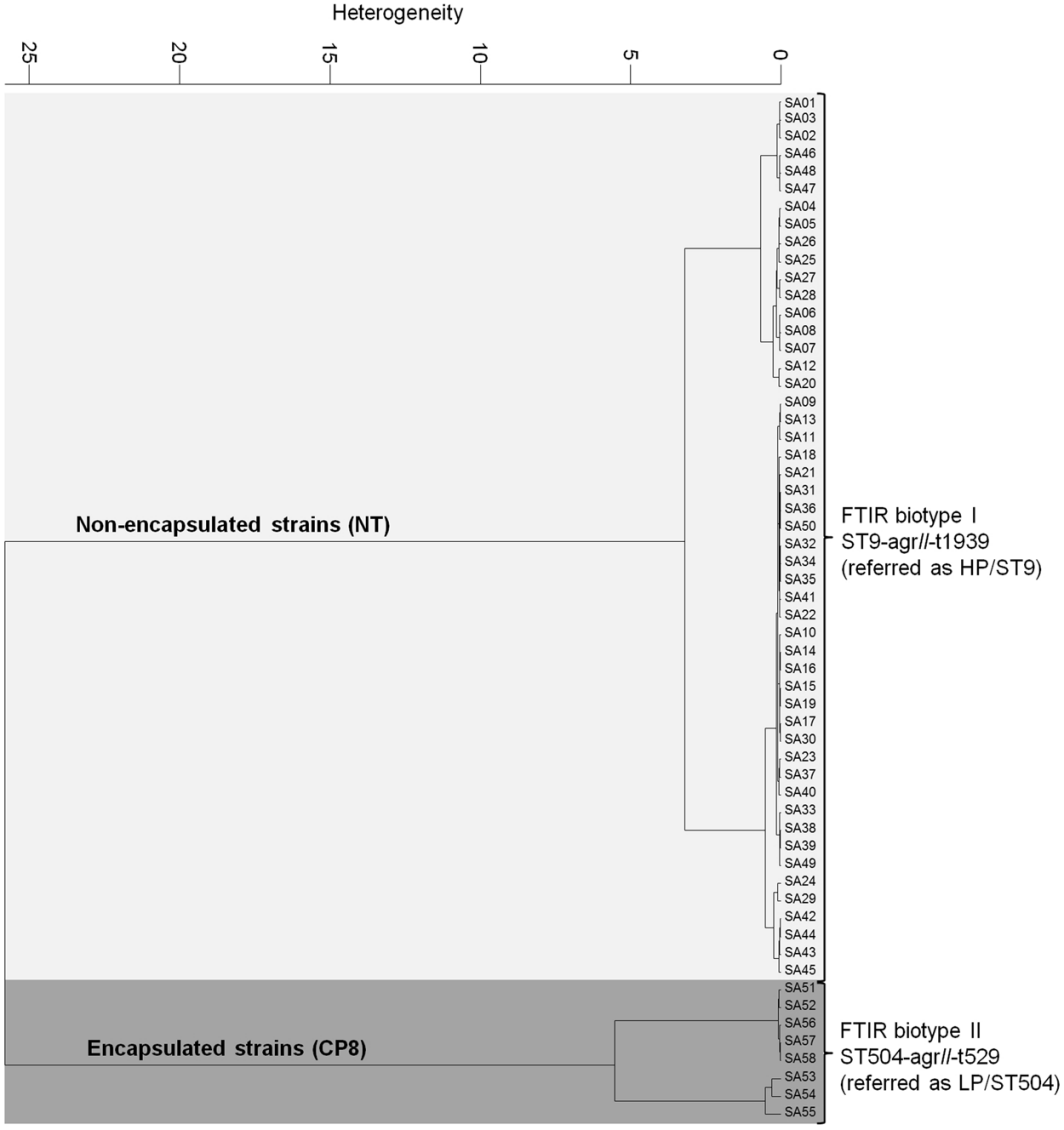
HCA dendrogam of FTIR biotyping. FTIR spectroscopy was employed to follow *S. aureus* persistence and spreading at herd level. In total two different FTIR biotypes were identified (I, II). The HP/ST9 isolates persisted and successively spread within the herd and did not express CP phenotypically, as they were defined as non-typeable (NT), whereas the LP/ST504 isolates expressed CP serotype 8 (CP8).

FTIR spectroscopy as a fast, high-throughput screening tool was initially used to subtype the herd-specific diversity of *S. aureus* strains and to follow potential spreading between cows^4,20^. Using hierarchical cluster analysis (HCA) of FTIR spectroscopic data we were able to discriminate two distinct FTIR biotypes (Fig. 1). The high IMI prevalent biotype I (n = 50; 86.2%) successfully persisted for years in several cows and spread at the herd level over the whole period infecting ten cows in total. Using molecular subtyping methods, this biotype could be assigned to *spa*-type t1939, ST9 [clonal-complex (CC) 9] and *agr*-II (hereinafter referred as high-prevalent/ST9, HP/ST9). The strains belonging to biotype II (n = 8, 13.8%) showed consistently lower within-herd prevalence. The latter biotype, which was isolated from the udder of two cows over a period of more than two years was assigned to *spa*-type t529, ST504 (CC705, former CC151) and *agr*-II (hereinafter referred as low-prevalent/ST504, LP/ST504). A summary and a detailed schema of isolates are provided in Table 1 and Suppl. 1, respectively.

**Table 1:** Summary of characteristics of mastitis derived strains used in this study.

FTIR biotype	MLST	*spa* type	IMI prevalence	Cows #^a^	*agr* type	CP type^b^	Virulence gene profile^c^	Toxin gene profile^d^	Referred as
I	ST9 (CC9)	t1939	50 (86,2%)	10	*II*	NT (*cap*5)	*ica*A, *ica*D, *fnbp*A*, clf*A*, clf*B	*seg*, *sei*	HP/ST9
II	ST504 (CC705)	t529	8 (13.8%)	2	*II*	CP8 (*cap*8)	*ica*A, *ica*D, *fnbp*A*, clf*A*, clf*B	*seg*, *sei*	LP/ST504

^a^Number of cows with pos. *S.aureus* IMI

^b^Capsular polysaccharide (CP) expression and presence of capsular polysaccharaide genes 5 and 8 (*cap5, cap8*).

^c^Presence of the intercellular adhesion genes A and D (*icaA, icaD*); fibronectin binding proteins A and B (*fnbpA, fnbpB*); clumping factor A and B genes (*clfA, clfB*).

^d^The presence of *S. aureus* enterotoxin genes (*sea, seb, sec, sed, see, seg, seh, sei, sej*) was tested.

Based on FTIR spectroscopic and genotypic subtyping, persistent mastitis isolates were divided into two groups related to either high- or low within-herd IMI prevalence. From each group, three *S. aureus* mastitis isolates derived from the last sampling time point of different animals were selected and further investigated regarding their properties associated with persistence, including cellular invasion, cytotoxicity and biofilm formation. The well documented and characterized bovine strain RF122 (ST504/ CC705)^21^, capable to reproducibly induce severe mastitis, was included as reference strain in this study (hereinafter referred RF122/ST504).

### HP/ST9 does not express a capsule

Since loss of capsular polysaccharide expression is thought to be associated with a chronic state of mastitis^14^, we utilized the recently developed CP typing system based on artificial neuronal network-assisted FTIR spectroscopy to identify capsule expressing (CP5, CP8) and non-expressing (non-typeable, NT) strains^22^, which was accompanied by c*ap* specific PCR determination. All HP/ST9 isolates expressed no CP and were found to be positive for the *cap5* specific allele*, w*hereas all LP/ST504 isolates expressed CP serotype 8 (CP8/ *cap8*). These results underpin that lack of CP expression is associated with high within-herd prevalence.

### HP/ST9 exhibits high internalization capacity on MAC-T cells

**Figure 2:**
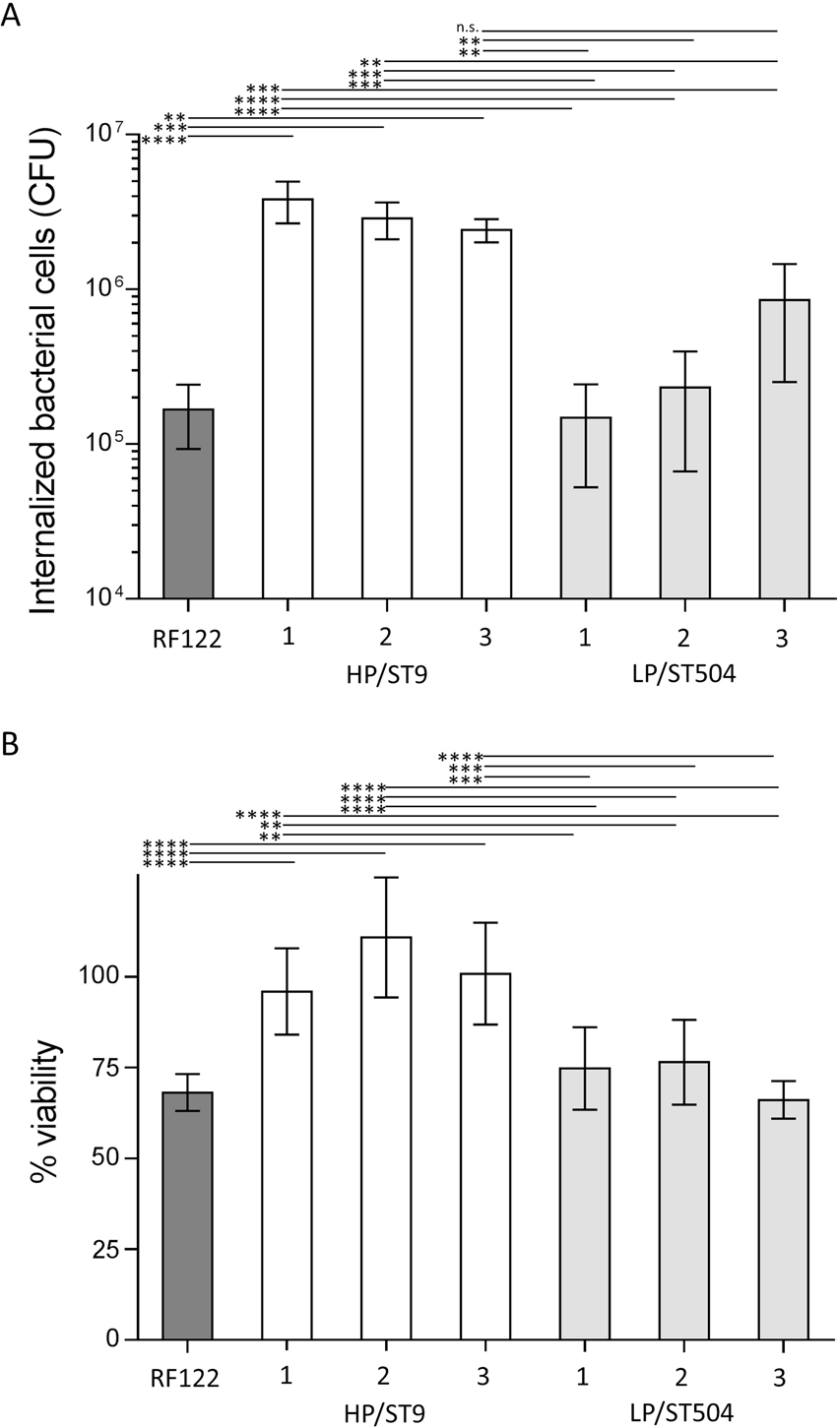
Bacterial internalization capacity and cellular cytotoxicity. From each group with either high or low within-herd IMI prevalence, HP/ST9 and LP/ST504, three different *S. aureus* mastitis isolates and the mastitis reference strain RF122 were comparatively tested. (**A**) *S. aureus* internalization into mammary epithelial (MAC-T) cells measured as CFU remaining after 2 h of incubation and killing of extracellular bacteria. (**B**) Cytotoxicity of *S. aureus* supernatants on MAC-T cells. The relative viability was expressed based on RPMI diluted in DMEM (ratio 1:1)-treated cells (=100%). Each bar represents the arithmetic mean ± standard deviation (SD) from three independent experiments. (*p < 0.05, **p < 0.01; ***p < 0.001; ****p < 0.0001).

Staphylococcal host cell invasion is considered to be one mechanism to establish a long-lasting, chronic infection^23^. Thus, we investigated the capacity of HP/ST9 and LP/ST504 isolates to internalize bovine epithelial cells *in* vitro, using bovine mammary epithelial (MAC-T) cells. MAC-T cells were incubated with *S. aureus* at a multiplicity of infection (MOI) of 10 to 50 for 1 h at 37 °C, and cells were subsequently washed and antibiotic-containing medium was added to eradicate the extracellular staphylococci. The high prevalent HP/ST9 showed a significant higher capacity to internalize epithelial cells compared to LP/ST504 and the bovine reference strain RF122/ST504. The internalization capacities of LP/ST504 isolates and strain RF122/ST504 showed no significant differences (Fig. 2A). Therefore, it is tempting to speculate that a high internalization capacity promotes staphylococcal high within-herd prevalence.

### HP/ST9 exhibits low cytotoxicity

The cytotoxic capacity of the isolates was examined in order to assess a possible correlation to within-herd prevalence. *S. aureus* cytotoxic effect was investigated by measuring the viability of MAC-T cells. Mitochondrial activity of RPMI-treated cells (100% viability) was used for normalization. HP/ST9 isolates showed only very low *in vitro* cytotoxicity. In contrast, LP/ST504 isolates and RF122/ST504 yielded a significantly stronger cytotoxicity than HP/ST9 (Fig. 2B).

### HP/ST9 produces more biofilm and higher amounts of PNAG than LP/ST504

**Figure 3:**
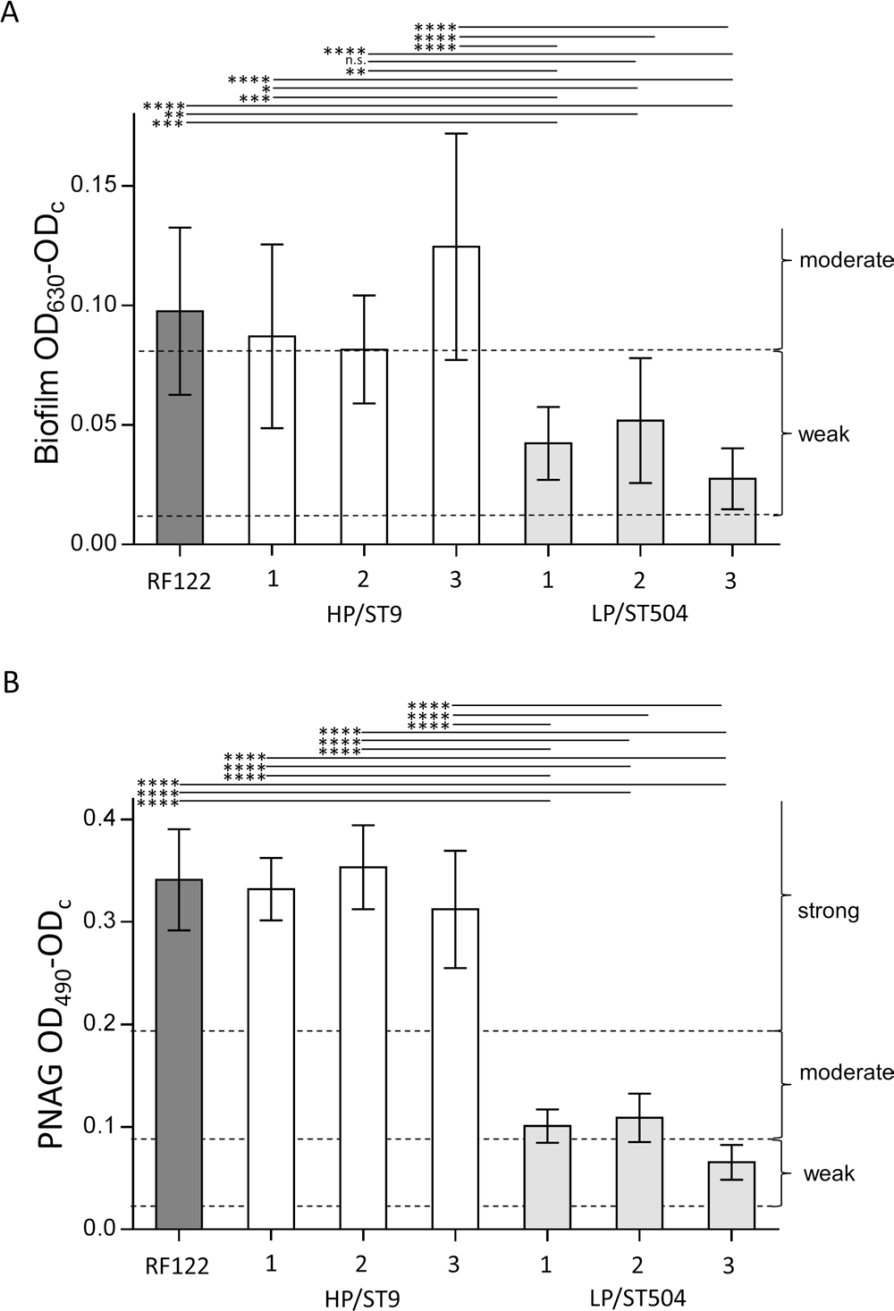
Biofilm production and PNAG content in biofilm. Biofilm (**A**) and PNAG in biofilm (**B**) was measured after 24 h growth in TSB media in a 96-well plate static biofilm assay. Un-inoculated medium served as a cut-off value (OD_c_) for the quantitative classification of biofilm and PNAG production in no, weak, moderate and strong producers based on their average OD values. Each bar represents the arithmetic mean ± standard deviation (SD) from three independent experiments. (*p < 0.05, **p < 0.01; ***p < 0.001; ****p < 0.0001).

Bacterial biofilms have been described to be associated with chronic bovine udder infections^5^, thus we next assessed whether high within-herd prevalent strains might be associated with an increased capacity to form biofilms *in vitro*, using a 96-well assay. In addition, the production of a major component of *S. aureus* biofilm the poly-N-acetyl-β-(1–6)-glucosamine (PNAG), also referred as extracellular matrix polysaccharide intercellular adhesin (PIA), was quantified using a lectin-based ELISA. HP/ST9 isolates turned out to produce moderate amounts of biofilm, but significantly more biofilm compared to LP/ST504 isolates, which are weak biofilm producers (Fig. 3A). Furthermore, the quantitation of PNAG expressed in biofilms revealed that HP/ST9 isolates are strong PNAG producers while LP/ST504 isolates are weak PNAG producers. The reference strain RF122/ST504 showed a biofilm/ PNAG phenotype similar to that of HP/ST9 isolates (Fig. 3B). These results indicate that biofilm production and particularly a high PNAG content in biofilms correlate with high within-herd prevalence.

### HP/ST9 and LP/ST504 isolates show no differences in their virulence gene and antibiotic resistance profiles

The presence of *S. aureus* adhesion (*fnbp*A, *fnbp*B, *clf*A, *clf*B), biofilm-associated (*ica*A, *ica*D) and *S. aureus* enterotoxin (SEs; *sea*, *seb*, *sec*, *sed*, *see*, *seg*, *seh*, *sei*, *sej*) genes was analyzed, using the panel of representative HP/ST9 and LP/ST504 strains, but no differences in virulence genes nor in the antibiotic resistance genes were observed between the high and low prevalent isolates. Both, high and low within-herd prevalent strains, were positive for *fnbp*A, *clf*A, *clf*B, *ica*A, *ica*D and *seg*, *sei*, (see also Table 1). In addition, antibiotic resistance was tested and revealed a broad susceptibility to the majority of tested antibiotics, but no distinct differences between the antibiotic resistant profiles between high and low prevalent isolates were found. Resistance against pirlimicin, erythromycin and marbofloxacin, but not against oxacillin (methicillin) for the high and low within-herd prevalent isolates was found. Antimicrobial resistance profiles are summarized in Table 2.

**Table 2:** MICs for selected antibiotics.

	Isolate	PEN	MIC (µg/ml)									
			AMP	CEZ	CPZ	CEQ	OXA	PIR	ERY	AMC	GEN	MAF
HP/ST9	1	0.125	4	4	2	1	1	2	4	4/2	2	0.5
	2	0.125	4	4	2	1	1	2	4	4/2	2	0.5
	3	0.125	4	4	2	1	1	4	1	4/2	2	0.25
LP/ST504	1	0.125	4	4	2	1	1	2	2	4/2	2	0.5
	2	0.125	4	4	2	1	1	1	2	4/2	2	0.25
	3	0.125	4	4	2	1	1	2	2	4/2	2	0.5

Resistance according to published breakpoints are underlined (see MM section). PEN, Penicillin G; AMP, ampicillin; CEZ, cefazolin; CPZ, cefaperazon; CEQ, cefquinom; OXA, oxacillin; PIR, pirlimycin; ERY, erythromycin; AMC, amoxicillin-clavulanic acid; MAF, marbofloxacin.

### HP/ST9 and LP/ST504 colonies show differences in formation of dendrites

**Figure 4:**
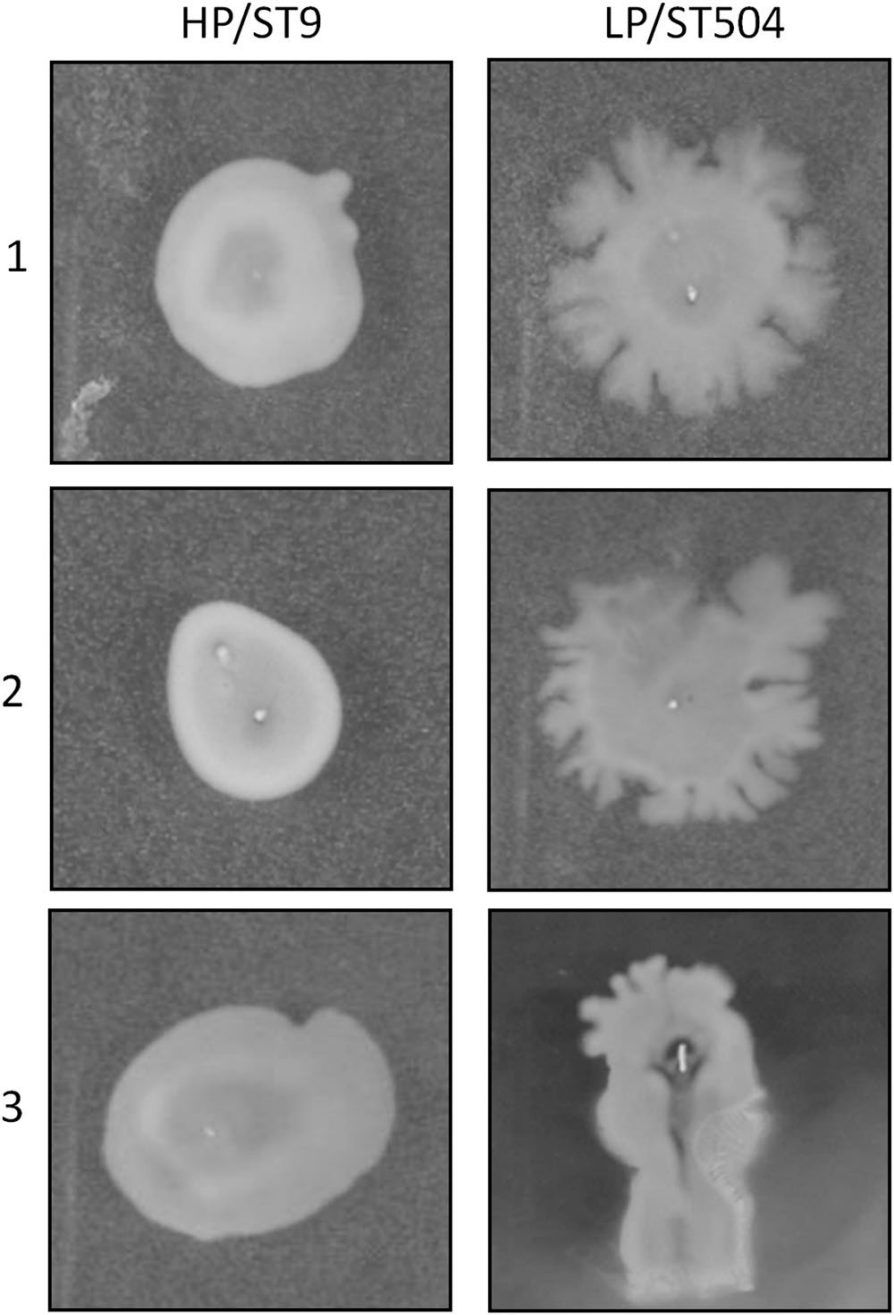
Expansion of bacteria on agar media. Bacterial cultures of HP/ST9 and LP/ST504 isolates were inoculated in the middle of the agar plate and subsequently incubated overnight at 37 °C. LP/ST504 formed colony dendrites on the agar media, whereas the HP/ST9 subtype not. Images are representatives from three independent experiments.

Furthermore, the formation of spreading dendrites has been linked to survival and spreading of *S. aureus* within host^24,25^. We therefore assessed the capacity of the high and low-prevalent isolates to spread on agar media. As depicted in Fig. 4, colonies of the low prevalent LP/ST504 showed formation of dendrites while no dendrite formation was observed in HP/ST9.

## Discussion

Several studies reported links between biofilm formation, CP expression and internalization capacity to chronicity in bovine IMI. However, these studies are based on well-established mastitis reference strains, such as RF122 and Newbould 305^26^, or on strains derived from different local or clonal (CC) origin^15,27^, while data from studies addressing differences of specific phenotypic and virulence traits of persistent strains in the context of within-herd prevalence are still lacking. To gain insights into the mechanism allowing *S. aureus* to spread and successfully persist within a herd, this study focuses on isolates collected over a 3-years period from a single dairy herd, which differed in their within-herd prevalence. Notable, all isolates could be assigned to two distinct biotypes and sequence types/ clonal complexes, namely HP/ST9 (CC9) and LP/ST504 (CC705), which were associated with high and low within-herd prevalence, respectively. Further analyses of representative strains revealed distinct phenotypic characteristics for both subtypes, which might at least partially explain their differences found for their prevalence on herd level.

Our study demonstrates that non-encapsulation and cellular invasion on bovine epithelial cells is associated with high within-herd prevalence of *S. aureus*. This is in line with previous findings that loss of CP expression is one key *S. aureus* feature associated with persistence^23^. It has been suggested that non-encapsulated *S. aureus* isolates might more effectively enter epithelial cells than encapsulated strains. Internalization might protect bacteria from clearance by the immune system and allow longtime persistence in chronically infected hosts^23^. Moreover, capsule-negative *S. aureus* strains have also been shown to induce chronic mastitis in a mouse model^14^ and the prevalence of non-encapsulated strains was reported to be higher in chronic than in acute infections in bovine mastitis as well as in human osteomyelitis and cystic fibrosis^11,28,29^.

Furthermore, reduced cytotoxicity was proposed to represent an additional mechanism contributing to persistence of staphylococcal infections^30^. This hypothesis is fostered by the results of our current study, showing that high within-herd prevalence is associated with a low cytotoxic potential. Contradictory, a higher potential cytotoxicity for high within-herd prevalent ST8 strains was proposed by a recent study of Capra *et al*.^31^. Thus, further studies will be necessary, including different ST types, before firm conclusion can be drawn on the importance and role of strain cytotoxicity for *S. aureus* persistence in dairy cattle.

Increased biofilm formation has been associated with chronic bovine udder infections and in line with this, we found higher levels of biofilm formation by strains with high within-herd prevalence. The same applies for the biofilm PNAG content, which mediates the bacterial intercellular connection and is produced by the *ica*ABCD operon encoded enzymes. In the present study, all biotypes were positively tested for *icaA* and *icaD* genes. These results support previous studies, showing a high prevalence of genes of the *icaA* and *icaD* loci among *S. aureus* mastitis isolates^32,33^. As reported for the clumping dispersal in *S. aureus* bloodstream infections^34^, high levels of PNAG in biofilm could be an advantageous dissemination mechanism by facilitating staphylococcal bacteria-to-bacteria interaction, which mediates the dispersal ability of the pathogen in the bovine mammary gland.

Based on the results of an *in vitro* agar plate assay, it has been discussed by Pollitt *et al*.^25,35^, that the formation of spreading dendrites might contribute to the establishment of *S. aureus* infections and translocation of the bacteria within the host. Since the low prevalent subtype LP/ST504 but not the high prevalent HP/ST9 subtype showed the formation of spreading dendrites in the agar plate assay, it is tempting to speculate that dendrite formation of the low prevalent *S. aureus* bovine mastitis isolates might be an adaptive feature to facilitate dispersal deep into the alveolar units of the mammary gland tissue fostering persistence within single animals rather than spreading within a herd.

Recent epidemiological studies showed that only the three clonal complexes CC8, CC97, and CC705, are largely dominating bovine IMI associated strains^36^. Isolates of ST504 (CC705) are known to frequently cause mastitis in cattle worldwide and these strains are described as archetypical bovine strains. In our study, LP/ST504 isolates were capable to persist for more than two years, but showed only limited success in spreading at herd level. This finding agrees with previous reports that CC705 strains exhibit only low contagiousness and are restricted to very few cows per herd resulting in low within-herd prevalence^16^. Notably, the isolates showing high within herd prevalence and persistence for years of our current study belong to ST9 (CC9). *S. aureus* ST9 methicillin- susceptible *S. aureus* (MSSA) and methicillin- resistant *S. aureus* (MRSA) strains are generally considered to exhibit their main reservoir in swine and also capable to colonize and infect humans^37^ and have so far only reported from some rare cases of bovine mastitis in Europe and China^36,38^.

## Conclusion

This study provides evidence for the existence of specific phenotypic traits linked to persistence of *S. aureus* in dairy cattle. Two persistent subtypes were found differing in their within-herd prevalence. In particular, the importance of lack of CP expression and high cellular invasiveness for *S. aureus* spreading within chronically infected dairy cattle herds is highlighted. Additionally, low level of cellular cytotoxicity and high biofilm/ PNAG production might support the capacity of strains to successfully persist and spread in bovines. Furthermore, first evidence is presented that the swine- and human-associated ST9 (CC9)-MSSA can also cause long-term persistent bovine IMI and is able to successfully spread between cows at the herd level, resulting in high within herd prevalence. This high dissemination potential of HP/ST9 discovered in our work renders dairy herds as a possible underestimated reservoir for human associated ST9 (MSSA/ MRSA) strains and thus, might represent a serious threat to public health, which requires further attention.

## Materials and Methods

### Sampling, isolation and identification of bovine isolates

*S. aureus* isolates were recovered from milk samples in frame of routine microbial mastitis diagnostics derived from cows (Simmental breed) with subclinical mastitis from a single dairy herd with 25 animals in average (tethered housing, manual milking stanchion) located at the provincial state Upper Austria over a period of more than three years (2010–2013). There was neither a study-related interference in any way with the herd management by the professional Veterinarian nor were bacteria experimentally administered to the cows. Subclinical IMI was characterized by milk somatic cell counts >200.000 cells/mL with no macroscopic changes following the guidelines of the National Mastitis Councils Laboratory Handbook on Bovine Mastitis^39^. Cases of subclinical, chronic, persistent *S. aureus* mastitis were regularly treated by antibiotics during dry-off. Milk samples were collected aseptically from foremilk and stored at −80 °C until bacteriological analysis^39^. Milk samples (approx. 10 ml) were centrifuged and a loop of sediment (10 μl) was streaked onto on Columbia agar supplemented with 5% sheep blood (CBA) (Thermofisher Scientific Inc., Oxoid Ltd., Hampshire, UK). CBA plates were incubated at 37 °C and examined after 24–48 hours for bacterial growth. Presumptive *S. aureus* isolates were confirmed by *S. aureus* species specific *nuc*-PCR^40^ and FTIR spectroscopy as mentioned below.

### Reference strains

Bovine strain RF122 (ST504/ CC705, former CC151), capable to reproducibly induce severe mastitis, was included as reference strain in this study^21^. *S. aureus* Reynolds prototype strain CP5 and its isogenic mutants Reynolds CP8 and Reynolds CP- (non-encapsulated)^41^ were used as controls for *cap* genotyping as well as for FTIR spectroscopic serotyping.

### Identification, subtyping and capsular serotyping by FTIR spectroscopy

Sample preparation and FTIR measurement was performed as described previously^22^. In brief, isolates were grown on tryptone soy agar (TSA) plates (Thermo Fisher Scientific, Oxoid) at 30 °C for 24 h. FTIR spectra of dried intact bacteria were recorded in transmission mode with an HTS-XT microplate adapter coupled to a Tensor 27 FTIR spectrometer (Bruker Optics GmbH, Ettlingen, Germany). Species identification was performed using a FTIR reference spectral library and confirmed by *nuc*-PCR^40^. *S. aureus* high-resolution strain typing by FTIR spectroscopy was performed as previously reported using average spectra of measurements performed on three different days^20^. Using the same spectra, capsular serotypes (CP5, CP8, NT) were determined by artificial neuronal network (ANN) analysis as previously established in our lab^22^.

### Molecular subtyping and virulence factor detection

MLST was performed according to Enright *et al*.^42^ with the exception of the downstream trimming position used to define *gmk* alleles, which has been adjusted to 417 bp, compared to the previous 429 bp^42^. The allelic profiles and ST were assigned based on the MLST website (http://saureus.mlst.net/). For *spa* typing the sequence of a polymorphic VNTR in the 3’ coding region of the *S. aureus*–specific staphylococcal protein A (spa) was used^43^ (www.spaserver.ridom.de). *Spa* types were assigned according to the repeat succession with the *spa*Typer free public application software (http://spatyper.fortinbras.us/). *Agr*-groups were determined by multiples PCR according to Gilot *et al*.^44^. The following genes were amplified by PCR: *ica*A/D^45^, *cap*5/8^46^, *fnbp*A/B^47^ and *clf*A/B^48^. *S. aureus* enterotoxin (SE) profiles were determined by PCR including *sea*, *seb*, *sec*, *sed*, *seg*, *seh*, *sei*, and *sej*^49^.

### Antibiotic resistance profiling

The antimicrobial resistance (AMR) was tested by applying the commercially available Micronaut-S Mastitis 2 MIC microtiter plate assay (Merlin; Sifin diagnostics GmbH, Berlin, Germany) including a panel of 10 antimicrobials: penicillin G, ampicillin, cefazolin, cefaperazon, cefquinom, oxacillin, pirlimycin, erythromycin, amoxicillin-clavulanic acid, and marbofloxacin. The breakpoints for minimum inhibitory concentrations (MICs) were determined according to the actual Eucast (http://www.eucast.org/clinical_breakpoints/) and Clinical and Laboratory Standards Institute (CLSI) standards^50^.

### Invasion assay

Bovine mammary epithelial cell line (MAC-T) was used for *S. aureus* internalization assay and was performed as previously reported with slight modifications^51^. Briefly, confluent MAC-T cell monolayers (~2 × 10^5^ cells/well) were inoculated with bacteria to produce multiplicities of infection (MOI) of 10–50. After incubation for 1 h at 37 °C under 5% CO_2_, the wells were washed with PBS and the invasion medium supplemented with gentamicin (final conc. 400 µg/mL) was added to each well to kill extracellular bacteria. After 1 h the monolayer was lysed by the addition of 0.025% Triton X-100 in sterile distilled water to release intracellular staphylococci. The CFU number was determined by quantitative plating on TSA agar. Three independent experiments on different days were performed each in triplicates per isolate.

### Cytotoxicity

*S. aureus* cytotoxic effect was assessed by measuring the viability of MAC-T cells based on tetrazolium salt WST-1 (Roche, Basel, Switzerland) to detect the mitochondrial activity of viable cells. Strains were grown under iron-depleted RPMI 1640 (Biochrome) to mimic mastitic conditions *in vivo*^52^. After 24 h of growth, the cultures were centrifuged and the supernatant was filtered on 0.22μm PVDF membrane units and subsequently diluted in DMEM at ratio 1:1. MAC-T cells were grown in this media for 24 h in 5% CO_2_ and the absorbance was read at 450 nm after adding WST-1 reagent for 60 min. All values were normalized to the cytotoxicity of cells treated with iron-depleted RPMI diluted in DMEM (ratio 1:1), which was set at 100% viability. Three independent experiments were performed in triplicates.

### Biofilm production ability and PNAG content

Biofilm formation was quantitatively assessed in a microtiter well plate assay using the CV staining method^53^. *S. aureus* overnight cultures were diluted 1:100 in trypticase soy broth (TSB, Oxoid) supplemented with 0.25% glucose (TSB-glucose) and transferred to 96-well polystyrene microtiter plates. After 24 h of static incubation at 37 °C, wells were washed, dried at 40 °C and the biofilm was fixed with methanol for 15 min prior staining with 0.1% CV. The quantity of biofilm biomass was determined by reading the optical density (OD) at 630 nm.

PNAG in biofilms was quantified according to Cramton *et al*.^54^ with slight modifications^54^. The biofilms for the PNAG assay were grown as described above. Biofilms were fixed by adding 95% ethanol for 15 min and blocked for 1 h at 37 °C in 1% w/v BSA in PBS plus 0.05% Tween 20 (PBST). After removal, wheat germ agglutinin horseradish peroxidase conjugate (WGA–HRP) (Sigma-Aldrich, St. Louis, MO, USA), was incubated for 30 min at 37 °C, stained with the peroxidase chromogenic substrate o-phenylenediamine (OPD) and subsequently measured at OD 490 nm. Un-inoculated medium served as a cut-off value (ODc) and the quantitative classification of biofilm production and PNAG content was based on average OD values according to Stepanovic *et al*.^55^. Three independent experiments were performed in at least quadruplicates for both assays.

### Motility assay

The motility assay was performed according to the detailed guidelines in Pollitt *et al*.^35^. In brief, an always fresh TSB media was prepared containing Bacto agar (0.34% in total), and D-Glucose (Sigma) (50 mM final conc.) was added after autoclave. Poured petri dishes were subsequently dried for 30 min in a safety cabinet and UV sterilized. Five µL of bacterial culture was spotted on the agar plate and plates were incubated overnight in a sealed plastic box at 37 °C.

### Statistical analysis

Statistical analysis was performed by using Graph-Pad Prism (GraphPad Software, Inc., La Jolla, USA; version 5.00). Statistically significant differences were calculated by using appropriate statistical methods as indicated including 1-way analysis of variance (ANOVA), followed by Dunnett’s multiple comparisons test for comparing the reference strain RF122 to the herd derived strains and the Sidak’s multiple comparisons test for comparing between high and low within-herd prevalent strains. *p* values less than 0.05 were considered significant and are indicated in the figures as follows: *p < 0.05, **p < 0.01; ***p < 0.001; ****p < 0.0001.

## Electronic supplementary material

Supplemantary Information

## Data Availability

The datasets generated and/or analyzed during the current study are available from the corresponding author upon request.
