# Supplementary material for: Distinct phenotypic traits of Staphylococcus aureus are associated with persistent, contagious bovine intramammary infections
Source: Sci Rep. 2018 Oct 29;8:15968. doi: 10.1038/s41598-018-34371-1 (PMC6206001; doi:10.1038/s41598-018-34371-1)
Supplement: Supplementary file 1 — Supplemantary Information [file 41598_2018_34371_MOESM1_ESM.pdf]

## Supplementary Information

# Distinct phenotypic traits of *Staphylococcus aureus* are associated with persistent, contagious bovine intramammary infections

Tom Grunert[1,*], Beatrix Stessl[2], Franz Wolf[3], Daniel O. Sordelli[4], Fernanda R. Buzzola[4], Monika Ehling-Schulz[1]

[1] Functional Microbiology, Institute of Microbiology, Department of Pathobiology, University of Veterinary Medicine, Vienna, Austria.

[2] Institute of Milk Hygiene, Milk Technology and Food Science, Department for Farm Animals and Veterinary Public Health, University of Veterinary Medicine, Vienna, Austria.

[3] Veterinarian Dr. Franz Wolf, Bad Schallerbach, Austria.

[4] Instituto de Microbiología y Parasitología Médica (IMPaM), Universidad de Buenos Aires and CONICET, Buenos Aires, Argentina.

Suppl. 1

Detailed list of mastitis isolates used in this study

| Within herd prevalence [a] | Isolate | Cow | Udder quarter | Year/ sampling point | FTIR biotype | CP type [b] | *spa* type | *agr* type | MLST | Isolates used for detailed examination |
|---|---|---|---|---|---|---|---|---|---|---|
| | SA01 | | RV | 1/1 | | | | | ST9/ CC9 | |
| | SA02 | | RV | 1/2 | | | | | | |
| | SA03 | | RV | 1/3 | | | | | | |
| | SA04 | | RV | 1/5 | | | | | | |
| | SA05 | | RH | 1/5 | | | | | | |
| | SA06 | | LH | 1/6 | | | | | | |
| | SA07 | 1 | LV | 1/7 | 1 | NT | t1939 | II | | |
| | SA08 | | RV | 1/7 | | | | | | |
| | SA09 | | RV | 2/1 | | | | | | |
| | SA10 | | LV | 2/1 | | | | | | |
| | SA11 | | LV | 2/2 | | | | | | |
| | SA12 | | LV | 3/1 | | | | | | |
| | SA13 | | RV | 3/1 | | | | | ST9/ CC9 | HP/ST9 - 1 |
| | SA14 | | RV | 1/1 | | | | | | |
| | SA15 | | LV | 1/2 | | | | | | |
| | SA16 | | RH | 1/2 | | | | | | |
| | SA17 | | RV | 1/2 | | | | | | |
| | SA18 | | LH | 1/7 | | | | | | |
| | SA19 | 2 | LV | 1/7 | 1 | NT | t1939 | II | | |
| | SA20 | | RH | 1/7 | | | | | | |
| | SA21 | | RV | 1/7 | | | | | | |
| | SA22 | | RV | 2/1 | | | | | | |
| | SA23 | | RH | 2/1 | | | | | | |
| | SA24 | | LH | 2/1 | | | | | | |
| | SA25 | | RH | 1/2 | | | | | | |
| | SA26 | | RH | 1/3 | | | | | | |
| | SA27 | 3 | RH | 1/6 | 1 | NT | t1939 | II | | |
| | SA28 | | RH | 2/1 | | | | | | |
| HP | SA29 | | RH | 2/1 | | | | | ST9/ CC9 | HP/ST9 - 2 |
| | SA30 | | LV | 2/1 | | | | | | |
| | SA31 | 4 | LV | 2/2 | 1 | NT | t1939 | II | | |
| | SA32 | | LV | 3/1 | | | | | | |
| | SA33 | | LH | 4/1 | | | | | | |
| | SA34 | | LV | 2/1 | | | | | | |
| | SA35 | 5 | LV | 2/2 | 1 | NT | t1939 | II | | |
| | SA36 | | LV | 3/1 | | | | | | |
| | SA37 | | LV | 4/1 | | | | | | |
| | SA38 | | RH | 1/4 | | | | | | |
| | SA39 | 6 | RH | 1/5 | 1 | NT | t1939 | II | | |
| | SA40 | | RV | 1/7 | | | | | | |
| | SA41 | | RH | 1/7 | | | | | | |
| | SA42 | | LV | 1/4 | | | | | | |
| | SA43 | 7 | LV | 1/5 | 1 | NT | t1939 | II | | |
| | SA44 | | LV | 1/6 | | | | | | |
| | SA45 | | LV | 2/1 | | | | | ST9/ CC9 | HP/ST9 - 3 |
| | SA46 | | RH | 1/6 | | | | | | |
| | SA47 | 8 | RH | 1/7 | 1 | NT | t1939 | II | | |
| | SA48 | | LV | 1/7 | | | | | | |
| | SA49 | 9 | LV | 1/6 | 1 | NT | t1939 | II | | |
| | SA50 | 10 | LV | 2/1 | 1 | NT | t1939 | II | | |
| | SA51 | | LH | 1/4 | | | | | ST504/CC705 | |
| | SA52 | | LH | 1/5 | | | | | | |
| | SA53 | 11 | LH | 1/7 | 2 | CP8 | t529 | II | | |
| | SA54 | | LH | 2/1 | | | | | ST504/CC705 | LP/ST504 - 1 |
| LP | SA55 | | LH | 2/3 | | | | | ST504/CC705 | LP/ST504 - 2 |
| | SA56 | | LH | 2/1 | | | | | | |
| | SA57 | 12 | LH | 2/2 | 2 | CP8 | t529 | II | | |
| | SA58 | | LH | 3/1 | | | | | ST504/CC705 | LP/ST504 - 3 |

[a] High (HP) and low (LP) prevalent isolates

[b] Capsular polysaccharide (CP) expression
